# 591. Positivity of Blood Culture on Automated Incubation System After 72 Hours and Clinical Significance from the Large United States Tertiary Care Center

**DOI:** 10.1093/ofid/ofad500.660

**Published:** 2023-11-27

**Authors:** Premalkumar M Patel, Cynthia Rivera

**Affiliations:** Mount Sinai Medical Center of Florida, Hollywood, Florida; Mount Sinai Medical Center, Miami Beach, Florida

## Abstract

**Background:**

Hospitals commonly use automated blood culture incubation systems that provide continuous monitoring for routine blood cultures. Detecting microorganisms early on can help optimize the utilization of antibiotics (ABx) and enable clinicians to de-escalate or discontinue ABx as necessary, making it an important aspect of antimicrobial stewardship. The BD BACTEC Fx, an automated system for blood culture incubation, holds blood culture bottles until growth is demonstrated or for five days unless an extended incubation period is requested by the infectious disease physician. We conducted a clinical evaluation of the significance of positive blood cultures after 72 hours of incubation time.

**Methods:**

After receiving approval from the institutional review board (IRB), we obtained positive blood cultures from July 2017 to June 2020, which we then stratified based on positivity from day one to day five. To evaluate clinical significance, we retrospectively reviewed charts for positive blood culture results on day four and day five. An Infectious Diseases subspecialty team determined the clinical significance of each case.

**Results:**

During the time frame of July 2017 to June 2020, there were 120,320 blood cultures taken. Out of these, 7,558 were determined to be positive, with 76 being positive on day 4 and 22 being positive on day 5. Upon further inspection of patients with positive blood cultures, it was discovered that repeat blood cultures continued to be positive due to either untreated sources or a high burden of bacteremia. It was also found that bottles containing fungus, particularly *candida glabrata* and *candida albicans*, tested positive on day 4 requiring treatment. Table 1 and Table 2 contain more detailed results.

Blood cultures positive on day 4

**Figure:**
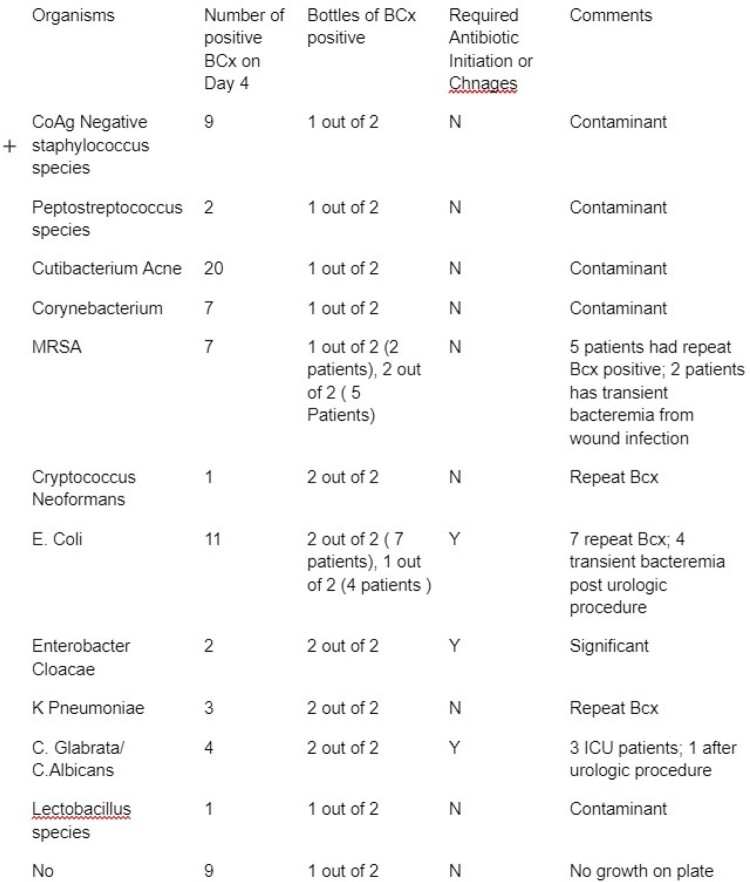

Blood cultures positive on day 4 and clinical significance

Blood cultures positive on day 5

**Figure:**
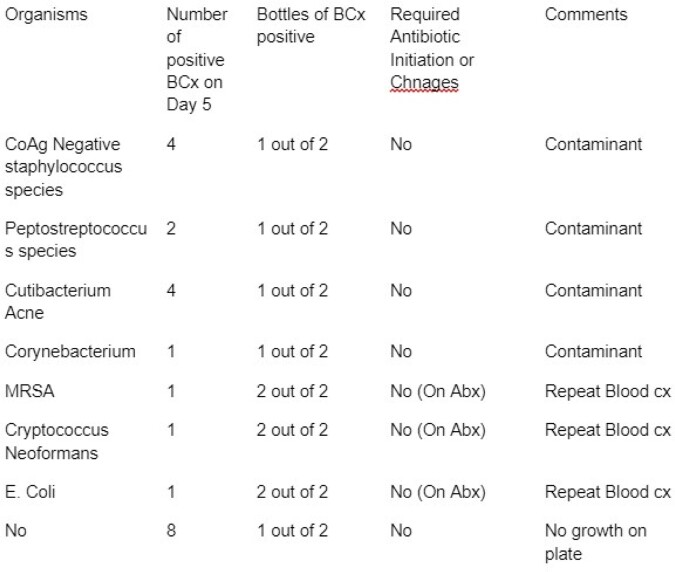

Blood cultures positive on day 5 and clinical significance

**Conclusion:**

In our study we have found that not all positive blood cultures at 72 hours translate into clinical scenarios requiring antibiotic therapy. In fact, prolonged antimicrobial treatment in such cases may lead to unnecessary exposure to antibiotics and increased risk of antibiotic resistance. It is important to consider the full context of clinical and laboratory findings when interpreting blood culture results, as well as any potential sources of contamination.

**Disclosures:**

**All Authors**: No reported disclosures

